# Cost-Effectiveness of Tenofovir Instead of Zidovudine for Use in First-Line Antiretroviral Therapy in Settings without Virological Monitoring

**DOI:** 10.1371/journal.pone.0042834

**Published:** 2012-08-08

**Authors:** Viktor von Wyl, Valentina Cambiano, Michael R. Jordan, Silvia Bertagnolio, Alec Miners, Deenan Pillay, Jens Lundgren, Andrew N. Phillips

**Affiliations:** 1 University College London, Research Department of Infection and Population Health, London, United Kingdom; 2 CSS Institute for Empirical Health Economics, Lucerne, Switzerland; 3 World Health Organization, Geneva, Switzerland; 4 Tufts University, School of Medicine, Boston, Masschussetts, United States of America; 5 London School of Hygiene and Tropical Medicine, London, United Kingdom; 6 University College London, Division of Infection and Immunity, London, United Kingdom; 7 Copenhagen University Hospital-Rigshospitalet, University of Copenhagen, Copenhagen, Denmark; Massachusetts General Hospital, United States of America

## Abstract

**Background:**

The most recent World Health Organization (WHO) antiretroviral treatment guidelines recommend the inclusion of zidovudine (ZDV) or tenofovir (TDF) in first-line therapy. We conducted a cost-effectiveness analysis with emphasis on emerging patterns of drug resistance upon treatment failure and their impact on second-line therapy.

**Methods:**

We used a stochastic simulation of a generalized HIV-1 epidemic in sub-Saharan Africa to compare two strategies for first-line combination antiretroviral treatment including lamivudine, nevirapine and either ZDV or TDF. Model input parameters were derived from literature and, for the simulation of resistance pathways, estimated from drug resistance data obtained after first-line treatment failure in settings without virological monitoring. Treatment failure and cost effectiveness were determined based on WHO definitions. Two scenarios with optimistic (no emergence; base) and pessimistic (extensive emergence) assumptions regarding occurrence of multidrug resistance patterns were tested.

**Results:**

In the base scenario, cumulative proportions of treatment failure according to WHO criteria were higher among first-line ZDV users (median after six years 36% [95% simulation interval 32%; 39%]) compared with first-line TDF users (31% [29%; 33%]). Consequently, a higher proportion initiated second-line therapy (including lamivudine, boosted protease inhibitors and either ZDV or TDF) in the first-line ZDV user group 34% [31%; 37%] relative to first-line TDF users (30% [27%; 32%]). At the time of second-line initiation, a higher proportion (16%) of first-line ZDV users harboured TDF-resistant HIV compared with ZDV-resistant viruses among first-line TDF users (0% and 6% in base and pessimistic scenarios, respectively). In the base scenario, the incremental cost effectiveness ratio with respect to quality adjusted life years (QALY) was US$83 when TDF instead of ZDV was used in first-line therapy (pessimistic scenario: US$ 315), which was below the WHO threshold for high cost effectiveness (US$ 2154).

**Conclusions:**

Using TDF instead of ZDV in first-line treatment in resource-limited settings is very cost-effective and likely to better preserve future treatment options in absence of virological monitoring.

## Introduction

The public health approach for combination antiretroviral therapy (cART) in resource-limited settings includes the use of one standard first-line and one standard second-line regimen [1]. According to World Health Organization 2010 treatment guidelines, first-line therapy should consist of a non-nucleoside reverse transcriptase inhibitor (NNRTI) and two nucleoside reverse transcriptase inhibitors (NRTI), one of which should be zidovudine (ZDV) or tenofovir (TDF). Second-line ART should consist of a ritonavir-boosted protease inhibitor (PI/r) plus two NRTIs, one of which should be ZDV or TDF, based on what was used in first-line therapy. Ritonavir-boosted atazanavir (ATV/r) or lopinavir/ritonavir (LPV/r) are the preferred PIs. The choice of using TDF or ZDV in first-line treatment is determined at country level. Randomized clinical trials have demonstrated superiority of TDF over ZDV [2], [3], [4], [5] and over stavudine (D4T) [6], [7] in combination therapy with regards to virological suppression, as well as a tendency for less toxicity-related discontinuations and improved adherence in industrialized [3] and resource-limited settings [8]. In contrast, the somewhat lower costs favour the use of ZDV, although considerable price reductions for TDF have been achieved more recently so differences are now small [9].

One particular concern regarding the widespread use of TDF in settings without virological monitoring is the potential for development of extensive nucleoside and nucleotide analogue cross-resistance via the emergence of the reverse transcriptase mutation K65R, and possibly also multidrug resistance patterns such as Q151M, although the latter has not been detected in well-controlled clinical trials in resource-rich settings [4], [7], [10]. Moreover, some in vitro data point to more rapid selection of K65R emergence in subtype C viruses, owing to a specific nucleotide motif at reverse transcriptase position 65 that facilitates the amino acid switch from lysine to arginine [11], [12]. Indeed, recent surveys from resource-limited settings suggest a comparatively high prevalence of high-level NRTI cross-resistance resistance associated with K65R (23%) or Q151M (0–19%) amongst patients with clinical or virological treatment failure [13], [14].

Previous cost effectiveness analyses have already pointed towards better clinical outcomes of TDF use compared with other NRTIs in industrialized [15] and resource-limited settings [16], [17], [18], [19]. These studies, however, mainly focused on HIV-1 and treatment related morbidities, and did not investigate the impact of the emergence of drug resistance mutations on future therapy options. In the present simulations, we aimed to re-assess the cost effectiveness of TDF over ZDV for settings using the public health approach for ART with one standard first-line and one standard second-line regimen, and without virological monitoring, which is the reality in most resource-poor settings. For this purpose, an established individual-based stochastic model of HIV transmission and treatment in a resource-limited country was adapted to reflect possible mutation patterns leading to and after first-line treatment failures and to predict costs of treatment for HIV-1 and tuberculosis-(TB) and HIV-related morbidity and mortality [20], [21]. We specifically considered the impact of the different resistance patterns generated by the use of TDF or ZDV in first-line cART on efficacy of second-line therapy and subsequent morbidity and mortality.

## Methods

### Stochastic Simulation

The model presented here corresponds to the version described extensively in [20], [21] and the accompanying web appendix (http://links.lww.com/QAD/A113), with deviations in how drug resistance mutations emerge (see below). In brief, the stochastic model, programmed in SAS 9.1, simulates a generalized heterosexual HIV epidemic in a resource-limited country by keeping track of individuals and their health status with regard to HIV and other co-morbidities. Individual characteristics are updated in three month time steps.

A typical simulation run, which is influenced by many random elements, shows the following characteristics: starting in 1989, the population of approximately 25 000 uninfected persons initially contains about 5 HIV infected individuals. The epidemic starts to spread via individuals who acquire HIV through heterosexual contacts with HIV-1 infected short or long term partners. The probability of transmission of HIV depends on whether the partner is undergoing primary infection, on the partner’s HIV-RNA viral load (obtained by sampling from the distribution of viral load levels found in partnerships formed by HIV-infected people, accounting for gender and age), on the subject’s gender and on the presence of other sexually transmitted infections. Each HIV-infected individual experiences HIV RNA levels, CD4 declines and mortality rates that correspond to their specific age and gender, health status with respect to co-morbidities, and to antiretroviral treatment exposure. We assumed that cART became available in 2007 (corresponding to the first availability of TDF in national and regional treatment programs [22], [23]), when the HIV prevalence had reached approximately 14%. Treatment either consists of fixed dose, twice daily ZDV+3TC+NVP or once daily TDF+ lamivudine (3TC) and NVP in two tablets, depending on the first-line treatment strategy.

Selected model input parameters are shown in Table 1. In the model, the number of active drugs, adherence and HIV RNA levels affect the probability for suppression of viral replication and the accumulation of resistance mutations. Each individual is assigned a fixed underlying adherence level, which can vary from period to period within certain bounds and can be offset (with an increment) in some circumstances according to specific rules (e.g. worse adherence when drug-related toxic side effects present, table 1). Following these fluctuations in adherence levels to antiretroviral drugs, HIV RNA can rise to detectable levels in individuals who receive ART. The risk for emergence of drug resistance follows an n-shaped relationship with adherence such that resistance risk is highest when adherence is moderate. Further details can be found in the Materials S1. When resistance has emerged, this reduces viral susceptibility to antiretroviral drugs and hence further reduces the probability for suppression of viral replication at the next time step.

**Table 1:** Selected model input parameters.

	Rates per 3 months	Value for sensitivity analysis	Source
Drug related toxicities (*1.5 times higher in first year)			
Zidovudine (ZDV)			
nausea*	0.1		own estimate
lipodystrophy	0.015		own estimate
anemia*	0.03		[3] and own estimate
Headache*	0.1		own estimate
lactic acidosis	0.001		own estimate
Tenofovir (TDF)			
Nephrotoxicity	0.01		[6] and own estimate.
**Antiretroviral treatment adherence**			
Adherence benefit of TDF over ZDV	0.03	0	[3]
worse adherence if drug related toxicities	0.1		[40]
**Resistance emergence (also see ** **figure 1** **)**			
Emergence of NNRTI mutations in presence of			
Detectable HIV RNA<500 copies/mL	0.4		own estimate
Detectable HIV RNA >500 copies/mL	0.95		own estimate
Emergence of M184V mutations in presence of			
Detectable HIV RNA<500 copies/mL	0.4		own estimate
Detectable HIV RNA >500 copies/mL	0.9		own estimate
**Other**			
Probability for switch if treatment failure detected	0.8	0.1	[41]

### Main Assumptions on Setting Characteristics

The setting of the main analysis consists of an HIV-infected population, in which ART has not previously been used. HIV diagnosis became available in 2003 and is either made by voluntary testing of a fixed rate of the population (7.5% chance every three months) or triggered by AIDS defining conditions. CD4 count determinations are performed every 6 months in diagnosed individuals, and if measured CD4 levels drop<200 cells/mm^3^ or a WHO stage 4 event has been diagnosed, ART is initiated.

The definition for treatment failure is based on clinical (new or recurrent WHO stage 4 condition), specific WHO clinical stage 3 conditions (e.g. pulmonary tuberculosis) and immunological criteria (fall of CD4 count to baseline or below, or 50% fall from on-treatment peak value, or persistent CD4 levels below 100 cells/mm3) and CD4 cell measurements occur on a 6-monthly basis. It is also assumed that virological testing is not available and that switching to second-line ART occurs almost immediately after detection of treatment failure at a rate of 80% per 3 months, unless an individual is lost to follow-up.

Second-line ART consists of LPV/r, 3TC, and either ZDV or TDF, whichever has not been used in first-line treatment already. In this hypothetical setting with only two lines of treatment available, individuals who fail second-line therapy remain on the failing regimen.

### Treatment Outcomes

Clinical (AIDS-defining conditions, mortality) and treatment outcomes (CD4 cell gain, rates of viral suppression, and treatment failure based on WHO criteria) of the simulation are presented as medians [2.5^th^–97.5^th^ percentiles] from the distribution of point estimates from all simulation runs per analysis (n = 100). Unless stated otherwise, treatment outcomes were estimated on an intent-to-continue treatment basis by the Kaplan-Meier method, meaning that study outcomes were still attributed to the respective first-line strategy in spite of possible switches to second-line therapy.

### Cost Analyses

Main cost-effectiveness outcomes are average costs (per treated individual) and cumulative costs accrued by year 2022 (15 years after cART became available) for antiretroviral treatment and expenses for management of TB or HIV-related morbidity. In addition, cumulative person-years and quality adjusted life-years (QALY) lived from ART start to death or until 2022, whichever came first, are compared between treatment strategies. On the basis of estimates from [24], we set utilities for estimation of QALY at 0.75 if drug-related toxicities were present, if the individual suffered from AIDS-defining conditions (ADC), or if the individual was infected with TB. Otherwise utility weights were set to 0.8 in HIV-infected individuals [24].

Costs for cART were derived from the Clinton Foundation price list of November 2010 [9]. The price per year of first-line treatment with ZDV/3TC/NVP and TDF/3TC/NVP was set at US$ 140 and US$ 147, respectively. Second-line therapy containing LPV/r and ZDV or TDF was priced at US$ 550. The per 3-month costs for management of TB, ADC, and *Pneumocystis carinii* prophylaxis (PCP) were US$ 50, US$ 200, and US$ 5, respectively. Costs for outpatient visits and laboratory monitoring (e.g. CD4 cell counts) were omitted, because they are the same for both treatment arms. As a measure of cost-effectiveness, incremental cost effectiveness ratios (ICER) were estimated. Incremental cost-effectiveness was defined as the difference in the average treatment costs per ART exposed individual between treatment strategies divided by the difference in average QALY per cART exposed individual between the two therapy strategies (ZDV first or TDF first) since cART became available. Thus, the ICER signifies the magnitude of additional costs incurred by the new treatment strategy to gain one additional QALY. Owing to the repetition of simulations, each model analysis yielded different predictions for QALYs and treatment costs. These results were summarized by calculating the ICER from averages of costs and QALYs over all simulations from the same setting/pathway. We further assessed the uncertainty of our model estimates. Because the simulation yielded no pairing of TDF and ZDV (the estimates for treatment arms were generated in separate simulation runs), we sampled one estimate from the TDF simulation and one estimate from the ZDV simulation to calculate the ICER. By repeating this procedure 1000 times we obtained a distribution of possible ICER outcomes, given the results from the 100 simulations per setting and scenario. We defined uncertainty bounds as the range that included 95% of all sampling repetitions. A health-care cost perspective was applied to the cost-effectiveness analysis, which had a time horizon of 15 years. Costs and life years lived were discounted at 3% per year. Cost effectiveness was determined according to WHO guidelines by comparing ICER estimates with the per capita gross domestic product (GDP; http://www.who.int/choice/costs/CER_levels/en/index.html *; WHO AFRO E region*). According to this definition, incremental cost effectiveness ratios below 3-fold the GDP (US$ 6461) are considered cost effective and ICER below the GDP (*US $2,154*) are very cost effective.

### Statistical Analyses of Genotypic Drug Resistance Tests to Derive Input Parameters for Drug Resistance Model

**Figure 1:**
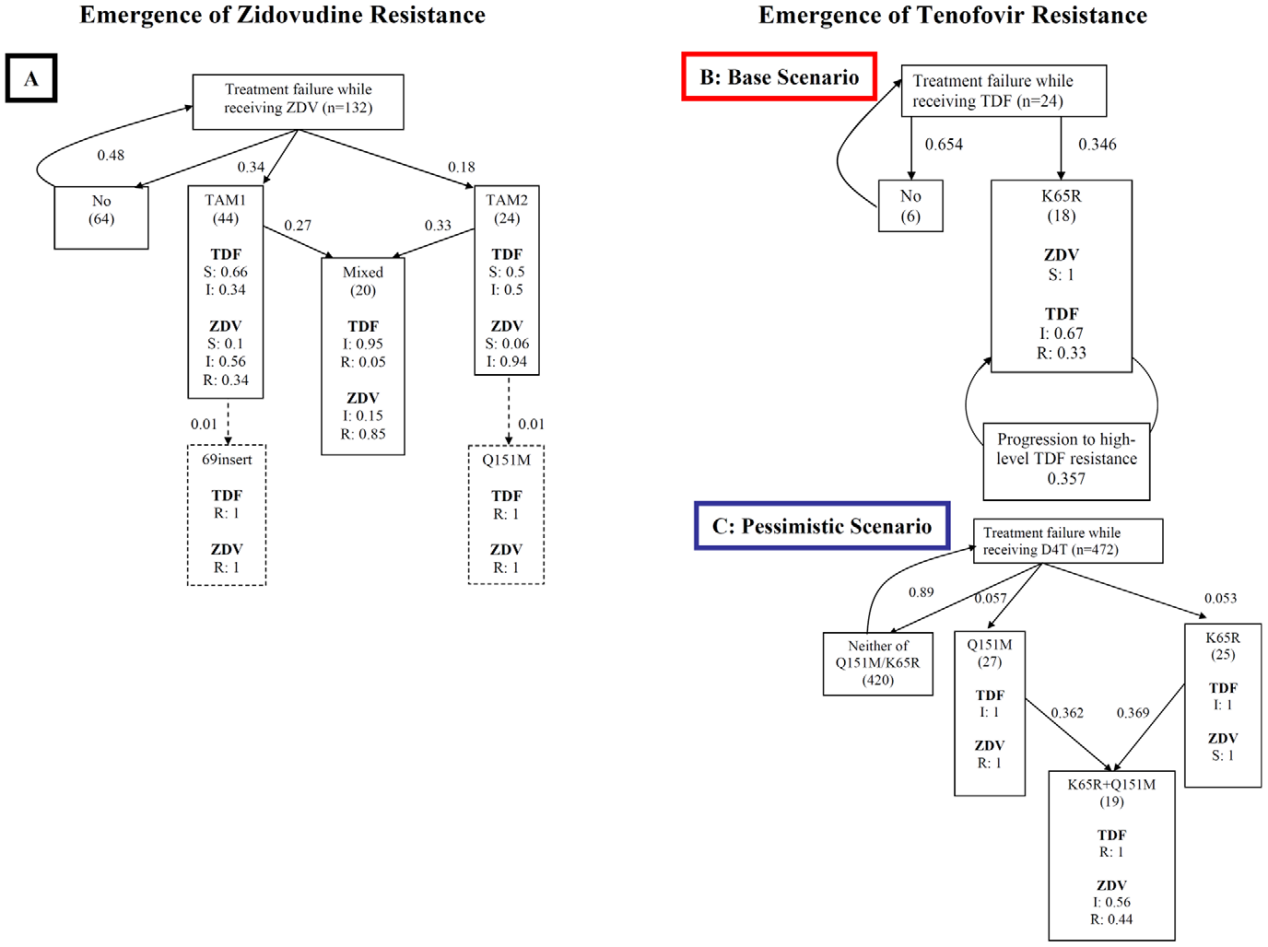
This plots hypothetical pathways of resistance emergence against zidovudine (1A) or tenofovir (1B & 1C) used in this simulation. The transition probabilities given next to arrows are per 3 months spent on a failing treatment with an (unmeasured) HIV RNA >500 copies/mL. Due to scarcity of resistance data of failing tenofovir regimens from developing settings two separate pathways were tested in the simulation. The base scenario (1B) was derived from a limited set of sequences from tenofovir failures and does not include the multidrug resistance pattern Q151M. The pessimistic scenario (1C) is based on estimations from sequences obtained after virological failure with stavudine and allows for extensive multidrug (i.e. Q151M) resistance emergence. Also note that the multidrug resistance patterns in the zidovudine pathway were not observed in the data (enframed by dashed lines), but were assumed to occur at low frequency. Abbreviations: ZDV, Zidovudine; TDF, tenofovir; S, susceptible; I, intermediate resistant; R, fully resistant.

The simulation modelled the emergence of thymidine analogue mutations (TAM), K65R, and Q151M on failing cART. To obtain estimates of rates and order of mutation accumulation of K65R and Q151M, descriptive statistical analyses of six publicly available data sets with genotypic drug resistance test (GRT) data of non-B subtype viruses from Sub-Saharan African settings were performed [13], [14], [25], [26], [27], [28]. Because the number of data points from first-line TDF use in resource-limited settings was very small (n = 24) in our sample, we additionally approximated rates of K65R and Q151M emergence from individuals failing first-line cART with D4T (along with lamivudine and an NNRTI), since this drug compound is also known to select for K65R and Q151M mutations and more data were available. Therefore, two separate simulations were run: A base scenario (Figure 1B) with mutation rates obtained from the limited set of sequences obtained from first-line TDF treated individuals, and a second scenario (Figure 1C) based on genotypic resistance test data collected after first-line treatment failure with D4T. Contrary to the base scenario, the pessimistic scenario allows for extensive emergence of the multidrug resistance pattern Q151M, hence it was termed the “pessimistic scenario”. Of note, TAM emergence was ignored in these two scenarios, because TDF does not select TAMs.

We constructed mutagenic trees by grouping the GRTs according to mutation patterns with respect to TAM, K65R, and Q151M, and by assuming a specific order for the emergence of these mutations (in Figures 1A, 1B, and 1C) [29]. This tree determines the order of mutation emergence (e.g. high emergence rates suggest early occurrence) as well as the progression of resistance along the tree in a time-dependent manner. Transition probabilities between tree nodes were estimated by counting the number of GRT showing a specific pattern and dividing them by the number of GRTs in the next higher tree node. These probabilities were converted into incidence rates per 3 months on a failing regimen by assuming an average time from virological treatment failure to stop or switch of the failing regimen of 1 year [14], [26], [27]. In addition, a genotypic sensitivity score (GSS) was estimated by applying the Stanford algorithm version 6.0.11 to each GRT that matched the mutation pattern of a specific tree node [30]. This procedure led, for each tree node, to a distribution of GRTs indicating full susceptibility, intermediate resistance, and full resistance to ZDV and TDF. In the stochastic simulation, progression in resistance pathways to the next node, as well as the degree of resistance were determined randomly, but corresponding to the probabilities observed at the respective node of the mutagenic tree.

The estimated 3-month incidence rates for NNRTI and 3TC mutations, which were also derived from genotypic data, are displayed in Table 1.

### Sensitivity Analyses

The effect of considering LPV/r worth only 1 drug, of immediate or delayed switching after detection of first-line treatment failure or of the assumed adherence benefit of TDF use on outcomes (and treatment costs in particular), and combinations of these parameters, were subjected to sensitivity analyses by re-running the simulation using predefined parameter values (Table 1). In the base scenario it was assumed that first-line TDF use led to a 3% point higher adherence compared with ZDV use due to better tolerability and once daily dosing [3].

Moreover, simulations were repeated in alternative settings, which included the availability of viral load monitoring, the effect of substituting drug components due to toxic side effects, and by introducing ART into a setting where transmitted drug resistance from D4T/3TC/nevirapine was present (for details see Materials S1) [31].

## Results

### Analysis of Observed and Predicted Drug Resistance Data

A total of 605 genotypic sequences obtained after first line treatment failure with either 3TC+ZDV+NNRTI (n = 133), 3TC+TDF+NNRTI (n = 24) or 3TC+D4T+NNRTI (n = 472) were analyzed. Given the small number of individuals who have received TDF in first-line treatment, rates of K65R and Q151M mutation emergence were also estimated from the D4T data and applied to a separate simulation representing an alternate, “pessimistic” scenario. The distribution of viral subtypes was as follows: C 53% (n = 320); G 16% (n = 97); CRF02_AG 14% (n = 83); and a variety of other non-B subtypes occurring at <4%.

Probabilities for the emergence of resistance upon clinical or immunological treatment failure were calculated as the percentage of genotypic resistance tests showing a specific mutation pattern and are displayed in Figure 1. When analyzing the D4T data, the K65R mutation emerged in 45 of 472 cases, which was considerably lower than what was seen in a limited sample of viral sequences from individuals with TDF treatment failure, where K65R was detected in 75% of 24 available genotypic sequences.

### Simulated Study Population at Time of ART Introduction

Out of a population of 4346 [4075; 4618] simulated HIV infected individuals, 2012 [1065; 2501] and 2045 [1536; 2517] individuals ever initiated ART with ZDV or TDF between the years 2007 and 2022 (end of simulation), respectively. The median age at time of cART initiation was 43 years, and 52% were women, irrespective of treatment strategy group. The median follow-up time after initiation of first-line therapy was 6 years. At time of therapy initiation, median values [interquartile range] of HIV RNA measurements were 5.09 [5.06; 5.12] log10 copies/mL, and median [interquartile] CD4 count measurements reached 140 [133; 147] cells/microliter, irrespective of treatment group. Around 7% [6]; [8] had active TB disease, and 9% [8]; [11] had experienced AIDS defining conditions.

Differences in first-line therapy outcomes were predicted with respect to CD4 cell count recovery, with a gain of 102 cells/microliter [97; 113] within 1 year in the ZDV group and gains of 114 cells/microliter [107; 121] (base scenario) and 107 cells/microliter [102; 112] (pessimistic scenario) in the TDF group, respectively. Intent to treat viral suppression rates below 50 copies\mL after 1 year were estimated at 64% [62; 67] among ZDV starters and at 68% [66; 71] (base scenario) and 66% [63; 68] (pessimistic scenario), respectively, in the TDF group.

**Figure 2:**
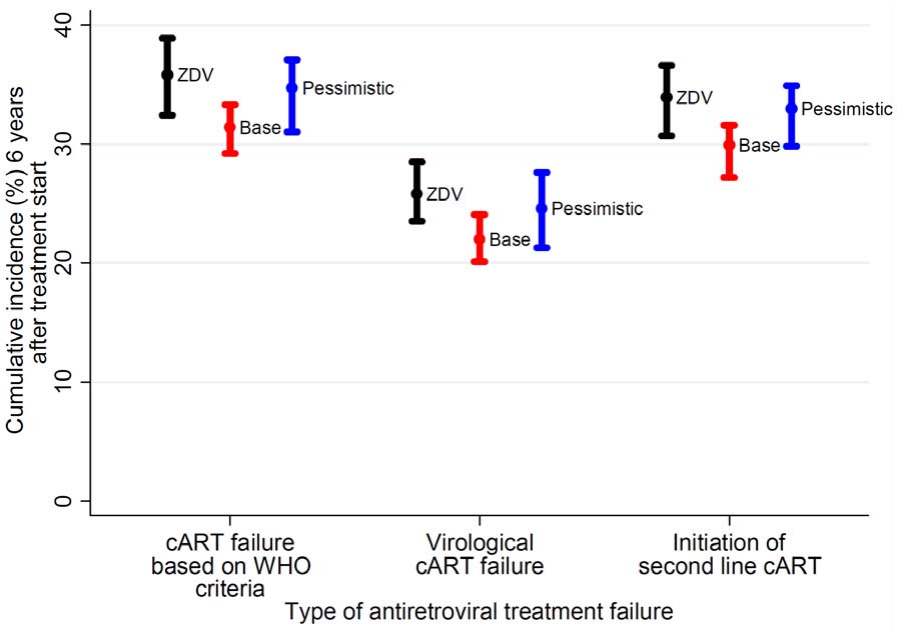
Shows different outcomes of first-line therapy by type of initial combination antiretroviral therapy (either including zidovudine [ZDV] or tenofovir [TDF]). For individuals starting with TDF, resistance emergence was modelled by two different scenarios (also see Figures 1B and 1C): a base scenario (red symbols) and a pessimistic scenario (blue symbols). Abbreviations: cART, combination antiretroviral therapy; WHO, World Health Organization.

As shown in Figure 2, six years after treatment initiation, corresponding to the median follow-up time after cART initiation, 25.8% [23.5; 28.5] of the ZDV starters had ever experienced a virological treatment failure, and 35.8% [32.4; 38.9] had experienced treatment failure according to the WHO definition based on immunological and clinical criteria. Among individuals who initiated treatment with TDF the proportions of virological and immunological/clinical failures were 22.0% [20.1; 24.1] and 31.4% [29.2; 33.3] respectively for the base scenario and 24.6% [21.3; 27.6] and 34.7% [31.0; 37.1] respectively for the pessimistic scenario. Six years after cART initiation, drug-resistance was predicted to have emerged in 27.9% [25.5; 30.4] of individuals in the ZDV group and in 24.3% [22.7; 26.1] (base scenario) and 26.4% [23.4; 29.8] (pessimistic scenario) of individuals in the TDF group.

### Predicted Patterns of Acquired Drug Resistance and Impact on Second-line Therapy

A higher proportion of ZDV starters was predicted to have initiated LPV/r-based second-line therapy within 6 years after antiretroviral treatment initiation (33.9% [30.7; 36.6], median n = 602) when compared with the group of TDF starters (base scenario: 29.9% [27.2; 31.6], median n = 547; pessimistic scenario: 33.0% [29.8; 34.9], median n = 597). Among individuals from the ZDV group who initiated second-line therapy, TAMs were predicted to be present in 28.4% [23.2; 32.5], and none in the two TDF groups. Among individuals who started TDF as first-line therapy, the predicted proportion of K65R was almost 9-fold higher in the base scenario (43.4% [38.8; 48.4]) compared with the pessimistic scenario (4.7% [3.3; 6.7]). In contrast, while there were no Q151M mutations emerging in the base scenario, the prevalence of Q151M was estimated at 5.9% [3.7; 8.3] in the pessimistic scenario. With respect to NNRTI mutations (56–57%) and M184V (62–63%), the simulation yielded almost identical estimates across the three groups (not shown).

Next, we analyzed the potential activity of second-line regimens against a background of different resistance mutations patterns. Previous studies have demonstrated that ritonavir-boosted PIs such as LPV/r have a very high potency to inhibit viral replication and are very robust to the emergence of drug resistance [32], [33]. Therefore, we allocated LPV/r a relative activity score of 1.5 in our analyses of second-line treatment outcomes. As shown in Table 2, when considering lopinavir/r as 1.5 active drugs and 3TC use in the presence of the mutation M184V as 0.25 active drugs (owing to the high viral fitness reduction induced by this mutation), 16.2% [12.3; 19.3] of ZDV starters were receiving second-line regimens with less than 2.75 active drugs (corresponding to partially active 3TC, a partially active NRTI, and fully active LPV/r). In contrast, depending on the scenario only 5.9% [3.7; 8.3] (pessimistic) or 0.6% [0; 1.6] (base) among the TDF starters received less than 2.75 fully active drugs. This marked difference was driven by ZDV’s potential to induce mutations, and in particular TAMs of group 1, with intermediate to full level cross-resistance to TDF (figure 1A). In contrast, K65R carrying viral strains are known to retain their susceptibility to ZDV [34]. The proportion of individuals with severely compromised second-line treatments with <2 fully active drugs (e.g. fully active LPV/r, partially active 3TC, and no activity of second NRTI) among ZDV starters was 0.7% [0.0; 1.3], but reached 5.0% [3.3; 7.8] when applying the pessimistic TDF scenario, which allows for the emergence of the multidrug resistance pattern Q151M. When translated into absolute numbers (per 1000 individuals starting first-line treatment) and taking into account first-line treatment failure rates, these predictions suggest that, six years after therapy start, 55 from the ZDV group and 19 from the TDF group (pessimistic scenario) will have started partially compromised second-line regimens with <2.75 fully active drugs. However, only 2 per 1000 individuals from the ZDV group, but 17 per 1000 from the TDF group (pessimistic scenario) will have initiated inadequate second-line therapy with <2 fully active drugs due to high-level cross-resistance.

**Table 2:** Percent of individuals starting partially inactive second-line treatment due to acquired drug resistance during first-line treatment.

Setting	ZDV first	TDF first, base scenario	TDF first, pessimistic scenario
Base			
<2.75 active drugs	16.2 [12.3; 19.3]	0 [0; 0.2]	5.9 [3.7; 8.3]
<2 active drugs	0.7 [0; 1.3]	0	5.0 [3.3; 7.8]
Transmitted Resistance			
<2.75 active drugs	16.3 [13.3; 20.6]	0.2 [0; 0.7]	6.2 [3.6; 8.9]
<2 active drugs	0.8 [0; 1.3]	0	5.4 [3.0; 7.6]
Virological Monitoring			
<2.75 active drugs	6.3 [4.8; 8.9]	0 [0; 0.1]	1.7 [1.0; 3.0]
<2 active drugs	0 [0; 0.5]	0	1.6 [1.0; 2.9]
Switches allowed			
<2.75 active drugs	14.3 [11.2; 18.8]	1.4 [0.4; 2.7]	6.5 [4.4; 8.9]
<2 active drugs	0.9 [0; 2.3]	0.8 [0.2; 1.7]	5.6 [3.1; 8.5]

### Cost Effectiveness Analysis

For the cost effectiveness analysis, the cumulative treatment costs accrued after therapy start until death or the year 2022 (whichever came first) were compared (table 3). The median observation time was 6 years, over which individuals starting therapy with TDF incurred slightly higher discounted ART-related costs (base scenario: US$ 1070; pessimistic scenario: US$ 1102) than individuals starting first-line treatment with ZDV with US$ 1058. However, less expenditures related to treatment of AIDS defining conditions or tuberculosis were needed in the group of TDF users (base scenario: US$ 148; pessimistic scenario: US$ 151) compared with the ZDV group with US$ 160 per individual on therapy.

**Table 3:** Cost effectiveness analyses.

Scenario	ZDV first, basescenario	TDF first, basescenario	ZDV first, pessimisticscenario	TDF first, pessimisticscenario
Base				
Treatment costs	1058 [904; 1145]	1070 [994; 1140]	1072 [995; 1148]	1102 [997; 1182]
HIV-morbidity related costs	160 [136; 170]	148 [135; 159]	160 [145; 170]	151 [136; 162]
Total costs	1217 [1040; 1314]	1219 [1137; 1292]	1232 [1143; 1314]	1252 [1138; 1338]
Life years lived	4.811 [4.290; 5.100]	4.936 [4.721; 5.172]	4.856 [4.642; 5.062]	4.910 [4.678; 5.125]
QALYs lived	3.719 [3.317; 3.944]	3.869 [3.701; 4.054]	3.753 [3.588; 3.917]	3.846 [3.663; 4.015]
* ICER for Treatment costs and*				
life years lived		99		540
QALYs lived		83		315
* ICER for total costs and*				
life years lived		11		377
QALYs lived		9		220
Transmitted Resistance				
Treatment costs	1076 [980; 1162]	1065 [827; 1135]	1070 [998; 1154]	1103 [950; 1177]
HIV-morbidity related costs	161 [151; 175]	149 [120; 159]	161 [145; 174]	152 [134; 162]
Total costs	1237 [1131; 1327]	1213 [948; 1289]	1231 [1146; 1326]	1255 [1085; 1339]
Life years lived	4.856 [4.519; 5.155]	4.895 [4.139; 5.161]	4.832 [4.551; 5.113]	4.891 [4.411; 5.129]
QALYs lived	3.754 [3.492; 3.986]	3.836 [3.244; 4.046]	3.735 [3.518; 3.955]	3.830 [3.454; 4.016]
* ICER for Treatment costs and*				
life years lived		dominant^a^		551
QALYs lived		dominant^a^		339
* ICER for total costs and*				
life years lived		dominant^a^		405
QALYs lived		dominant^a^		250
Virological Monitoring				
Treatment costs	1102 [1011; 1170]	1073 [918; 1152]	1112 [997; 1187]	1134 [1056; 1209]
HIV-morbidity related costs	150 [132; 161]	138 [113; 150]	149 [136; 161]	142 [132; 150]
Total costs	1252 [1151; 1325]	1211 [1031; 1295]	1261 [1135; 1339]	1277 [1188; 1355]
Life years lived	4.910 [4.558; 5.174]	4.977 [4.406; 5.243]	4.929 [4.576; 5.178]	4.995 [4.736; 5.241]
QALYs lived	3.800 [3.530; 4.001]	3.905 [3.456; 4.112]	3.815 [3.544; 4.008]	3.915 [3.712; 4.109]
* ICER for Treatment costs and*				
life years lived		dominant^a^		348
QALYs lived		dominant^a^		229
* ICER for total costs and*				
life years lived		dominant^a^		243
QALYs lived		dominant^a^		160
Switches allowed				
Treatment costs	1012 [952; 1080]	1021 [937; 1085]	1027 [962; 1087]	1054 [945; 1122]
HIV-morbidity related costs	164 [148; 175]	154 [139; 167]	163 [156; 172]	155 [144; 165]
Total costs	1176 [1104; 1253]	1175 [1084; 1244]	1190 [1126; 1250]	1209 [1091; 1287]
Life years lived	4.803 [4.543; 5.029]	4.908 [4.605; 5.126]	4.855 [4.662; 5.072]	4.884 [4.445; 5.128]
QALYs lived	3.723 [3.524; 3.899]	3.843 [3.606; 4.016]	3.763 [3.611; 3.935]	3.822 [3.477; 4.012]
* ICER for Treatment costs and*				
life years lived		86		937
QALYs lived		75		453
* ICER for total costs and*				
life years lived		dominant^a^		663
QALYs lived		dominant^a^		321

Footnotes:

All costs are in US$.

a TDF dominant over ZDV because of lower costs and higher QALYs.

**Figure 3:**
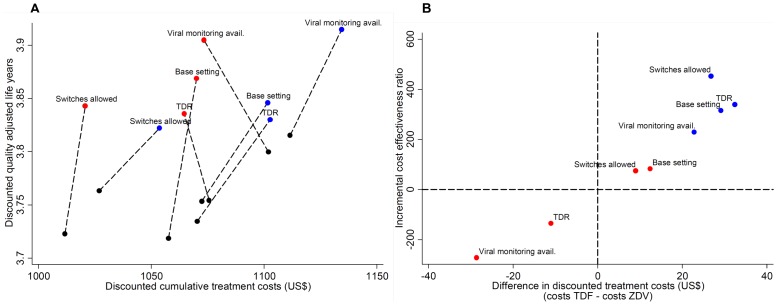
**Figure 3A** plots the average cumulative costs incurred by first-line tenofovir or zidovudine against quality adjusted life years. The connected dots refer to one set of comparisons between first-line tenofovir (TDF; coloured dots) or zidovudine (ZDV; black dots) using different assumptions regarding scenarios of TDF resistance emergence (red dots: base scenario; blue dots: pessimistic scenario involving the emergence of the Q151M multidrug-resistance complex; see Figure 1 for further details), as well as for alternative settings, which were the availability of 6-monthly HIV RNA determinations; >0% prevalence of transmitted drug resistance (TDR) in the study population; or exchange of ZDV with TDF (and vice versa) due to toxic side effects allowed (see Materials S1 for further details). Figure 3B plots estimates for incremental cost effective ratios against estimates for cumulative cost differences between first-line TDF and ZDV use.

Regarding morbidity and life-years lived as outcomes, the three strategies seemed to be comparable: the six year Kaplan-Meier estimates for mortality were 25.9% [22.1; 28.4] for the ZDV group and 23.3% [20.8; 25.9] (base scenario) and 23.6% [21.5; 25.4] (pessimistic scenario) for the TDF groups. The mean number of discounted life years lived since therapy start until death or 2022 per individual looked similar across the three groups (4.9 years, Table 3), but QALY gained were somewhat higher among first-line TDF users compared with the ZDV group (Figure 3A). Costs and QALY measures were then combined into incremental cost effectiveness ratios for different outcomes (Table 3, Figure 3B). When focusing on resistance emergence in the base scenario, our simulation results suggests that TDF use for first-line therapy was a very cost-effective treatment strategy, with an additional quality adjusted life year costing less than US$ 100, and in two scenarios (transmitted resistance present; availability of virological monitoring) even being a dominant strategy because of lower costs and more QALYs gained. However, when considering the pessimistic scenario, which allows for extensive NRTI multidrug resistance, the price for an additional quality adjusted life year rose to up to US$ 450 and TDF use was no longer dominant, although still very cost-effective by WHO standards.

Next, we assessed the robustness of model outcomes in different settings (presence of transmitted resistance, virological monitoring available, drug switches due to drug toxicities allowed; see Materials S1 for further details) and the reliance on the choice of specific input parameter values, namely the assumed better adherence to TDF compared with ZDV and the impact of delayed switching of drugs after detection of treatment failure. When considering the impact of different settings on model results (Figure 3), we observed that the availability of virological monitoring (i.e. 6-monthly HIV RNA determinations) generally improved cost effectiveness of first-line TDF use relative to the base setting. The other two changes to settings (i.e. presence of transmitted drug resistance or the option to switch drugs in case of toxic side effects) only had a limited effect on cost effectiveness outcomes.

Furthermore, the sensitivity of results to specific parameter values was explored (Table S1 and Table 4). In general, QALY estimates varied notably across the different sensitivity analyses. But overall cost-effectiveness of the TDF strategy over the ZDV strategy was maintained, and generally ICER estimates came to lie below the WHO cost-effectiveness threshold for high cost effectiveness (i.e. ICER < the per capita GDP) in ≥95% of cases (table 4).

**Table 4:** Uncertainty bounds of incremental cost effectiveness (ICER) estimates, % of ICER estimates suggesting dominance of the tenofovir (TDF) treatment strategy and % of ICER estimates below the WHO threshold for high cost-effectiveness.

Scenario/Sensitivity analysis	Base Scenario			Pessimistic Scenario		
	Uncertaintybounds ofICERestimates	% TDF strategy dominant	% ICERestimates< WHOthreshold	Uncertaintybounds of ICERestimates	% TDF strategy dominant	% ICERestimates< WHOthreshold
Base	[−2186; 3269]	27%	97%	[−2439; 2729]	9%	96%
Transmitted Resistance	[−3327; 3353]	32%	95%	[−1347; 2874]	10%	97%
Virological Monitoring	[−5970; 3747]	47%	96%	[−1576; 2683]	14%	97%
Switches allowed	[−1512; 3132]	23%	96%	[−2761; 2676]	6%	97%
LPV worth only 1 drug instead of 1.5	[−2222; 1901]	31%	98%	[−4490; 2133]	19%	98%
Median time to switch 22 months (i.e.3-month switch probability of 10%)	[−5111; 4950]	32%	95%	[−2981; 3111]	28%	96%
No additional adherence benefit for TDF	[−766; 1807]	5%	98%	[−1240; 2287]	8%	96%
Median time to switch 22 months & noadditional adherence benefit for TDF	[−1558; 2406]	13%	97%	[−5426; 5132]	30%	95%

Results were obtained by repeatedly drawing one simulation with TDF as the initial strategy and one simulation with zidovudine (ZDV) in the initial treatment. From this pair of simulations the incremental cost effectiveness ratio was calculated. Dominance was defined by lower costs and higher quality adjusted life year estimates for a specific treatment. By repeating this process 1000 times we obtained an estimate for how frequently the TDF strategy was dominant. Analogous calculations were performed to check how often the ICER estimates were below the WHO threshold for high cost effectiveness (annual per capita gross domestic product of US$ 2154). Uncertainty bounds reflect ranges that include 95% of all ICER estimates.

Abbreviations: ICER, incremental cost effectiveness ratio; TDF, tenofovir; WHO, World Health Organization.

## Discussion

By using an established stochastic simulation of HIV disease progression and therapy we have explored the impact of using different NRTI drugs (namely ZDV versus TDF) on resistance emergence and its consequences in terms of response to available second-line regimens and associated costs. Owing to uncertainty with respect to the influence of prolonged exposure to failing regimens and the effect of non-B subtype infection on NRTI-cross resistance we tested two pathways for resistance emergence while receiving TDF therapy. The base scenario assumed a rapid and frequent emergence of the TDF signature mutation K65R but only a very limited degree of NRTI cross-resistance. The second, pessimistic scenario was derived from analyses of genotypic resistance tests performed after failure of first-line combination treatment with D4T and was characterized by a more limited emergence of K65R, but a considerable risk for NRTI-cross resistance by the emergence of Q151M.

Our analyses suggest that first-line TDF use is a cost-effective treatment strategy compared with first-line ZDV use when considering quality adjusted life years as outcome, although dominance of the TDF strategy was only observed in 11% to 46% of comparisons (table 4). The use of TDF instead of ZDV also led to a reduction in treatment failures on the basis of WHO criteria by approximately 1% (pessimistic scenario) to 4% (base scenario). Consequently, fewer individuals in the TDF group had to switch to more costly second-line therapy compared with ZDV starters, although the magnitude of this difference was dependent on assumptions regarding the TDF resistance pathway. Our study results are line with those from a modelling analysis by Bendavid et al., who obtained an ICER estimate of US$ 1045 for first-line regimens consisting of TDF, 3TC and NVP when compared with first-line ZDV, 3TC and NVP [19]. Other published cost-effectiveness analyses are not directly comparable to our study, because their reference scenarios involved receiving no cART [16] or receiving D4T [18]. Nevertheless, both studies also reached the conclusion that the TDF first strategy may be cost-effective when compared with the ZDV first strategy because of better tolerability. Further support for this conclusion stems from analyses of antiretroviral treatment programs in southern Africa, which observed fewer drug related toxicity events among first-line TDF users when compared with individuals starting therapy with ZDV [22], [23]. In particular, severe TDF-associated renal toxicity was shown to be rare and often transient, and therefore does not seem to pose a major obstacle for widespread TDF implementation in settings without creatinine clearance monitoring [23], [35], [36]. In comparison, life-threatening anaemia or lipoatrophy occur frequently in association with ZDV-use, especially in malnourished populations [22], [23]. All these are drug side effects, which are not caused by TDF.

Depending on the actual rate of NRTI multidrug resistance emergence, first-line TDF use may increase emergence of extensively NRTI class-resistant HIV by 8.5-fold (17/1000 first-line TDF users in the pessimistic scenario compared with 2/1000 first-line ZDV users). Observational studies have reported associations of K65R mutations with Q151M, possibly pointing towards a co-selection of these mutations [37], [38]. However, these studies were performed among patients with extensive antiretroviral drug histories-including exposure to D4T or didanosine, but not necessarily TDF-, and the drugs responsible for selection of K65R and Q151M could not be determined with certainty. In contrast, currently available resistance data from individuals undergoing long-term therapy with TDF support the more optimistic scenario [10], [14], [39]. If true, the limited degree of cross-resistance even after extended exposure to failing treatment would make TDF a valid option for second-line therapy, in which EFV is replaced by LPV/r. A recent observational study suggests that in salvage settings staying on TDF may be preferable over switching to ZDV due to better tolerability and similar viral load reductions [34].

Some limitations should be noted about this study. Like any model, our simulation involves simplifications of reality and is based on assumptions regarding input parameters. In particular, given the lack of real data we had to make assumptions regarding rates and extent of drug resistance following immunological failures in resource-limited settings, as shown in Figure 1. Given these limitations, we subjected several important parameters to sensitivity analyses and repeated the simulation for different settings. We observed that the pessimistic simulation scenario with regards to drug resistance emergence reduces TDF cost-effectiveness, and so did changes to settings or other input parameters of interest (adherence levels, switch rates, and potency of LPV/r). But these results did not alter our conclusions, because TDF remained very cost effective by WHO standards (table 4). These analyses further revealed that a strategy of first-line TDF use in settings with virological monitoring would further enhance cost effectiveness relative to first-line ZDV use (Table 3 and Figure 3). It should also be noted that measures of treatment outcomes in our analysis such as the proportion of individuals with undetectable viral loads or the increase in CD4 cell counts from baseline tend to be somewhat lower than those observed in clinical trials and observational studies, although this finding has no direct impact on the cost-effectiveness analyses.

In summary, taking into account the possibility of more extensive drug resistance or possible long term renal toxicity by TDF use we conclude that first-line TDF use is likely to be a very cost-effective treatment strategy in resource-limited settings even in the absence of virological monitoring, because of the better tolerability and the small cost difference.

## Supporting Information

Table S1 **Cost effectiveness results from sensitivity analyses. Numbers in brackets represent 95% simulation intervals, i.e. the range including 95% of all model predictions.**
(DOC)

Materials S1 **Description of settings for main simulations and details on modelling of resistance-adherence relationships.**
(DOC)
