# Supplementary material for: Cost-Effectiveness of Tenofovir Instead of Zidovudine for Use in First-Line Antiretroviral Therapy in Settings without Virological Monitoring
Source: PLoS One. 2012 Aug 8;7(8):e42834. doi: 10.1371/journal.pone.0042834 (PMC3414499; doi:10.1371/journal.pone.0042834)
Supplement: Table S1 — Cost effectiveness results from sensitivity analyses. Numbers in brackets represent 95% simulation intervals, i.e. the range including 95% of all model predictions. (DOC) [file pone.0042834.s001.doc]

Table S1: Cost effectiveness results from sensitivity analyses. Numbers in brackets represent 95% simulation intervals, i.e. the range including 95% of all model predictions.

| **Sensitivity analyses** | **ZDV first, base scenario** | **TDF first, base scenario** | **ZDV first, pessimistic scenario** | **TDF first, pessimistic scenario** |  |
| --- | --- | --- | --- | --- | --- |
| LPV worth only 1 drug instead of 1.5 | |  |  |  |  |
| Treatment costs | 1037 [978; 1094] | 1039 [897; 1097] | 1045 [979; 1110] | 1057 [972; 1136] |  |
| HIV-morbidity related costs | 170 [157; 177] | 150 [136; 161] | 167 [154; 181] | 151 [141; 160] |  |
| Total costs | 1207 [1140; 1267] | 1188 [1032; 1251] | 1211 [1136; 1290] | 1208 [1112; 1291] |  |
| Life years lived | 4.776 [4.632; 4.943] | 4.885 [4.401; 5.085] | 4.818 [4.482; 5.089] | 4.890 [4.607; 5.116] |  |
| QALYs lived | 3.689 [3.576; 3.816] | 3.828 [3.449; 3.983] | 3.722 [3.460; 3.933] | 3.832 [3.610; 4.011] |  |
|  |  |  |  |  |  |
| *ICER for Treatment costs and* |  |  |  |  |  |
| life years lived |  | 15 |  | 171 |  |
| QALYs lived |  | 12 |  | 111 |  |
| *ICER for total costs and* |  |  |  |  |  |
| life years lived |  | dominant ^a^ |  | dominant ^a^ |  |
| QALYs lived |  | dominant ^a^ |  | dominant ^a^ |  |
|  |  |  |  |  |  |
| Median time to switch 22 months (i.e. 3-month switch probability of 10%) | | |  |  |  |
| Treatment costs | 1076 [970; 1152] | 1069 [988; 1145] | 1067 [888; 1167] | 1077 [1006; 1156] |  |
| HIV-morbidity related costs | 163 [148; 173] | 149 [138; 163] | 159 [138; 171] | 148 [134; 164] |  |
| Total costs | 1239 [1118; 1318] | 1218 [1134; 1308] | 1226 [1026; 1335] | 1224 [1141; 1320] |  |
| Life years lived | 4.885 [4.481; 5.122] | 4.933 [4.669; 5.254] | 4.838 [4.209; 5.092] | 4.962 [4.679; 5.216] |  |
| QALYs lived | 3.775 [3.462; 3.960] | 3.866 [3.661; 4.118] | 3.740 [3.258; 3.939] | 3.889 [3.668; 4.090] |  |
|  |  |  |  |  |  |
| *ICER for Treatment costs and* |  |  |  |  |  |
| life years lived | - | dominant ^a^ | - | 77 |  |
| QALYs lived | - | dominant ^a^ | - | 64 |  |
| *ICER for total costs and* |  |  |  |  |  |
| life years lived | - | dominant ^a^ | - | dominant ^a^ |  |
| QALYs lived | - | dominant ^a^ | - | dominant ^a^ |  |
|  |  |  |  |  |  |
| No additional adherence benefit for TDF | |  |  |  |  |
| Treatment costs | 1064 [996; 1135] | 1108 [1015; 1157] | 1054 [837; 1129] | 1098 [1042; 1166] |  |
| HIV-morbidity related costs | 160 [147; 171] | 151 [145; 159] | 159 [127; 169] | 152 [138; 160] |  |
| Total costs | 1225 [1145; 1306] | 1259 [1162; 1312] | 1213 [964; 1298] | 1250 [1189; 1316] |  |
| Life years lived | 4.831 [4.501; 5.058] | 4.929 [4.582; 5.147] | 4.786 [4.188; 4.977] | 4.880 [4.633; 5.073] |  |
| QALYs lived | 3.734 [3.480; 3.907] | 3.860 [3.586; 4.033] | 3.698 [3.239; 3.844] | 3.822 [3.630; 3.975] |  |
|  |  |  |  |  |  |
| *ICER for Treatment costs and* |  |  |  |  |  |
| life years lived |  | 446 |  | 464 |  |
| QALYs lived |  | 345 |  | 354 |  |
| *ICER for total costs and* |  |  |  |  |  |
| life years lived |  | 352 |  | 394 |  |
| QALYs lived |  | 272 |  | 301 |  |
|  |  |  |  |  |  |
| Median time to switch 22 months & no additional adherence benefit for TDF | | | |  |  |
| Treatment costs | 1069 [1010; 1172] | 1093 [1042; 1138] | 1084 [1016; 1112] | 1085 [1060; 1138] |  |
| HIV-morbidity related costs | 159 [151; 171] | 151 [142; 159] | 161 [155; 165] | 150 [140; 159] |  |
| Total costs | 1228 [1170; 1342] | 1244 [1189; 1294] | 1245 [1175; 1276] | 1235 [1204; 1290] |  |
| Life years lived | 4.860 [4.624; 5.143] | 4.912 [4.724; 5.136] | 4.866 [4.630; 4.987] | 4.871 [4.779; 5.020] |  |
| QALYs lived | 3.758 [3.570; 3.975] | 3.848 [3.700; 4.025] | 3.760 [3.578; 3.850] | 3.816 [3.744; 3.933] |  |
|  |  |  |  |  |  |
| *ICER for Treatment costs and* |  |  |  |  |  |
| life years lived |  | 463 |  | 207 |  |
| QALYs lived |  | 264 |  | 19 |  |
| *ICER for total costs and* |  |  |  |  |  |
| life years lived |  | 309 |  | dominant ^a^ |  |
| QALYs lived |  | 176 |  | dominant ^a^ |  |

Footnotes:

^a^: TDF dominant over ZDV because of lower costs and higher QALYs.
