# Supplementary material for: Cost-Effectiveness of Tenofovir Instead of Zidovudine for Use in First-Line Antiretroviral Therapy in Settings without Virological Monitoring
Source: PLoS One. 2012 Aug 8;7(8):e42834. doi: 10.1371/journal.pone.0042834 (PMC3414499; doi:10.1371/journal.pone.0042834)
Supplement: Materials S1 — Description of settings for main simulations and details on modelling of resistance-adherence relationships. (DOC) [file pone.0042834.s002.doc]

**Supplementary materials S1.**

*Description of settings for main simulations:*

Virological monitoring

In this setting, HIV RNA measurement is performed every six months, and treatment failure is defined in individuals with viral load >500 copies HIV RNA/ml after a minimum of six months continuous exposure to antiretroviral therapy. Consequently, treatment failures are detected much earlier than with monitoring based on clinical criteria alone.

Switches because of drug related toxicities allowed

In case of ZDV- or TDF- related drug toxicities, the substitution with the other component is allowed. Among ZDV starters, the cumulative incidence switch to TDF reached 9%, 15%, and 20% after 1, 2, and 3 years, respectively. In contrast, switches from TDF to ZDV occurred in <2% because severe drug related toxicities to TDF are rare.

Presence of transmitted drug resistance

This setting assumes that combination treatment with D4T, 3TC, and NVP has previously been used in the population, and consequently transmitted drug resistance mutations are present in the population. For each individual newly starting therapy in the simulation, a random number generator decides whether the individual is infected with drug resistant HIV. The following prevalence estimates for specific transmitted mutations are assumed [1]:

Thymidine analogue mutations: 1.4%; M184V: 1%; K65R: 0.4%; Q151M: 0.1%, or any non-nucleoside reverse transcriptase inhibitor mutations: 3.4%. These mutations can reduce viral susceptibility to first-line antiretroviral therapy.

*Details on the model of resistance-adherence relationships*

In the model, the number of active drugs, adherence and HIV RNA levels affect the probability for suppression of viral replication and the accumulation of resistance mutations.

In the beginning, each individual is assigned a fixed underlying adherence level, which can vary from period to period within certain bounds.

| **Distribution** | **Intrinsic Adherence** | **Variability** |
| --- | --- | --- |
| 5% | 50% | 20% |
| 10% | 80% | 20% |
| 25% | 90% | 6% |
| 60% | 95% | 5% |

Adherence at any one period is determined as follows (although with modifications explained below): adh(t) = *Intrinsic Adherence* + Normal**Variability*,

with Normal being the standard normal distribution.

Adherence can be offset (with an increment or decrement) in some circumstances according to specific rules, which are:

- Better adherence when TDF is used instead of ZDV in combination with lamivudine/nevirapine: +3% points
- Worse adherence in presence of ART-related side effects: -10% points.

Following fluctuations in adherence levels to antiretroviral drugs, HIV RNA can rise to detectable levels in individuals who receive ART. The risk for emergence of drug resistance follows an n-shaped relationship with adherence such that resistance risk is highest when adherence is moderate (Supplementary Figure 1). When resistance has emerged, this reduces viral susceptibility to antiretroviral drugs and hence further reduces the probability for suppression of viral replication at the next time step.

These estimates are based partially on observed adherence data [2,3], but also on adherence levels required to produce observed estimates of rates of resistance development and virologic failure and also data on the proportion of patients at first virologic failure who have no resistance mutations present [4].

Supplementary Figure 1: Resistance-Adherence relationship in the model

References:

1. Hamers RL, Wallis CL, Kityo C, Siwale M, Mandaliya K, et al. (2011) HIV-1 drug resistance in antiretroviral-naive individuals in sub-Saharan Africa after rollout of antiretroviral therapy: a multicentre observational study. Lancet Infect Dis. 11(10):750-9.

2. Mills EJ, Nachega JB, Buchan I, Orbinski J, Attaran A, Singh S et al. (2006) Adherence to antiretroviral therapy in sub-Saharan Africa and North America - A meta-analysis. JAMA; 296 (6): 679-690.

3. Bangsberg DR, Moss AR, Deeks SG et al. (2004) Paradoxes of adherence and drug resistance to HIV antiretroviral therapy. J Antimicrob Chem; 53 (5): 696-699.

4. Mackie N, Phillips AN, Kaye S, et al. (2010) Antiretroviral drug resistance in HIV-1 infected patients with low-level viraemia. J Infect Dis; 201:1303-1307
